# Thymic lipofibroadenoma of the anterior mediastinum: A rare case report

**DOI:** 10.1097/MD.0000000000031732

**Published:** 2022-11-18

**Authors:** Jing Fu, Xing-Wei Cai, Shuang-Ye Hu, Tao Lu, Xing-Lan Li

**Affiliations:** a Department of Pathology, Sichuan Provincial People’s Hospital, University of Electronic Science and Technology of China, Chengdu, Sichuan Province, China; b Chinese Academy of Sciences Sichuan Translational Medicine Research Hospital, Chengdu, Sichuan Province, China; c Department of Anesthesiology, Chengdu First People’s Hospital, Chengdu, Sichuan Province, China; d Department of Pathology, Longquanyi District of Chengdu Maternal and Child Health Care Hospital, Chengdu, Sichuan Province, China; e Department of Radiology, Sichuan Academy of Medical Sciences & Sichuan Provincial People’s Hospital, Chengdu, Sichuan Province, China.

**Keywords:** anterior mediastinum, case report, differential diagnosis, lipofibroadenoma, thymic tumor

## Abstract

Lipofibroadenoma is an extremely rare thymic tumor, and the anterior mediastinum is the most common site.

**Case summary:**

A 21-year-old male was admitted with fever without obvious cause for 2 months. After admission, the patient’s highest temperature was 38.3°C, accompanied by diarrhea. Physical examination showed coarse breath sounds in both lungs. Chest enhanced computed tomography (CT) showed a mass of mixed density shadow on the left side of the anterior mediastinum with a size of approximately 9.2 cm × 5 cm × 2.1 cm and a clear boundary mixed with a low fat density shadow. Mediastinal tumors were removed under general anesthesia by video-assisted thoracoscopic surgery. Macroscopically, a clear boundary was shown between the tumor and the remaining thymus. Microscopically, the tumor contained a large amount of mature adipose and fibrous tissue with scattered cord-like epithelial tissue and a small number of lymphocytes scattered in the stroma. The tumor lacked thymic bodies. The neoplastic epithelial cells were oval or polygonal and arranged in fissures, the nuclei were uniform in size and mild in shape, and mitosis was rare. Epithelial cells were positive for AE1/AE3 and CK19, lymphocytes were positive for CD3 and CD20, and fat and fibrous tissue were positive for S-100 and vimentin, respectively. The Ki67 labeling index was less than 5%. Based on histological features and immunophenotype, thymic lipofibroadenoma was diagnosed. The patient was followed up 1 year after the operation, and no recurrence or residual lesions were found on the X-ray re-examination.

**Conclusion:**

Lipofibroadenoma is a benign thymic tumor, and thymectomy is regarded as the best treatment. The biological behavior of thymic lipofibroadenoma is good, and the recurrence rate is low.

## 1. Introduction

Lipofibroadenoma is an extremely rare thymic tumor with morphologic characteristics similar to breast fibroadenoma, accounting for 6% to 17% of all thymic tumors. Lipofibroadenoma usually occurs in patients between 40 and 50 years old and affects men and women equally.^[1–3]^ Similar to the classic B1-type thymoma, the anterior mediastinum is the most common site of lipofibroadenoma. Histopathology shows that the tumor consists of a large amount of mature adipose tissue and a large number of broad bands of dense fibrous tissue with scattered bands of epithelial tissue. The tumor cells are oval or polygonal, and the nuclei are mild and uniform in size. Thymus bodies are rare in this neoplasm. The biological behavior of thymic lipofibroadenoma is good, and the recurrence rate is less than 10%.^[4]^ Thymic tumor resection is the preferred treatment. Here, we report a case of thymic lipofibroadenoma and briefly review the literature.

## 2. Case presentation

### 2.1. Clinical data

**Figure 1.**
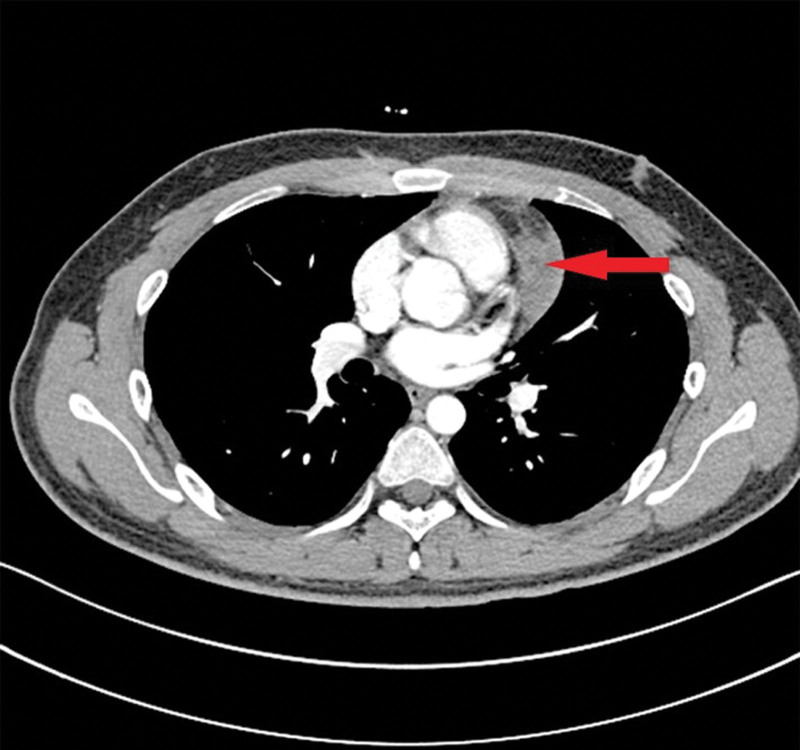
Imaging: Chest enhanced CT showed a mass of mixed density shadow on the left side of the anterior mediastinum. CT = computed tomography.

A 21-year-old male was admitted with fever without obvious cause for 2 months. After admission, the patient’s highest temperature was 38.3°C, accompanied by diarrhea. The patient did not have cough, sputum, chest tightness, chest pain, dizziness, drooping eyelids, limb weakness or dyspnea. Physical examination showed coarse breath sounds in both lungs, and no rales were detected. Chest enhanced computed tomography (CT) showed a mass of mixed density shadow on the left side of the anterior mediastinum with a size of approximately 9.2 cm × 5 cm × 2.1 cm and a clear boundary mixed with a low fat density shadow (Fig. 1). Uneven enhancement was revealed on enhanced CT. Lipofibroadenoma was considered during imaging diagnosis. Mediastinal tumors were removed under general anesthesia by video-assisted thoracoscopic surgery.

### 2.2. Pathological data

The samples included the tumor and surrounding thymus tissue. The tumor was oval in shape, 8 cm × 6 cm × 2 cm in volume, solid in section, gray in color and hard in quality. Specimens were cut along the longitudinal axis and fixed with 10% neutral formalin buffer for 12 hours. Fifteen representative tissue blocks from different regions were successively dehydrated and embedded in paraffin. The section thickness was 4 μm.

**Figure 2.**
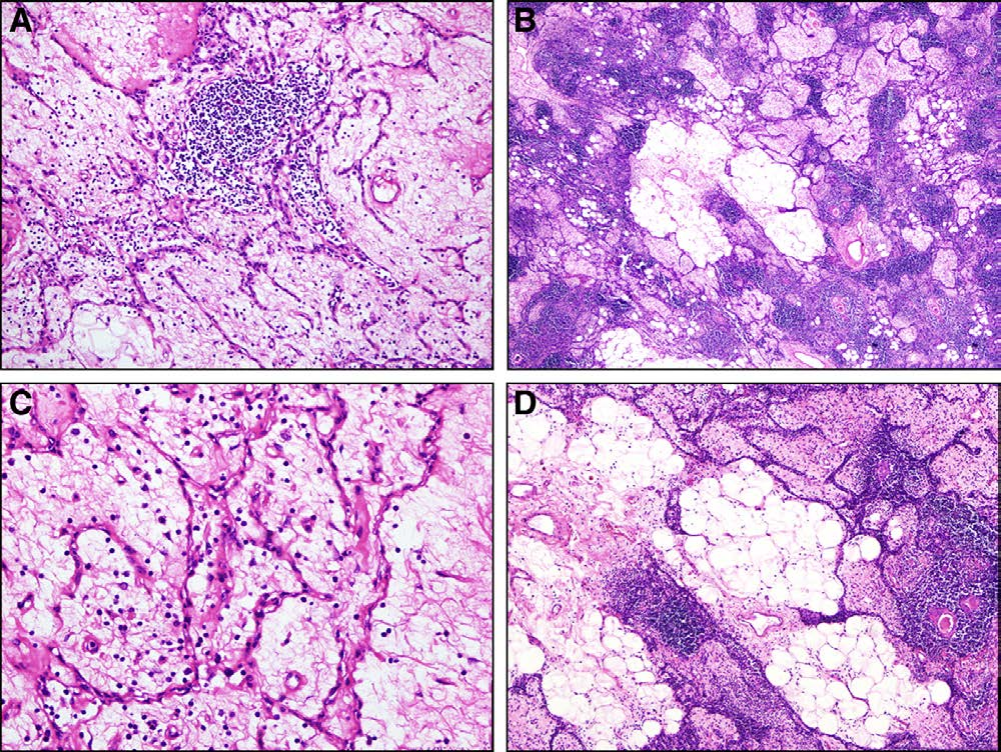
Histological examination of lipofibroadenoma. (A&C) The tumor was observed with irregularly connected figurate strands of thymic epithelial cells in a fibrous tissue, the elongated epithelial were recorded to be formed various structures and sparse lymphocytes were infiltrated. (B&D) The fat cell was distributed singly or multifocally in the fibrous tissue. (H&E staining, A&B: ×100, C&D: ×400).

Macroscopically, a clear boundary was shown between the tumor and the remaining thymus. Microscopically, the tumor contained a large amount of mature adipose and fibrous tissue with scattered cord-like epithelial tissue and a small number of lymphocytes scattered in the stroma (Fig. 2). The tumor lacked thymic bodies. The neoplastic epithelial cells were oval or polygonal and arranged in fissures, the nuclei were uniform in size and mild in shape, and mitosis was rare.

**Figure 3.**
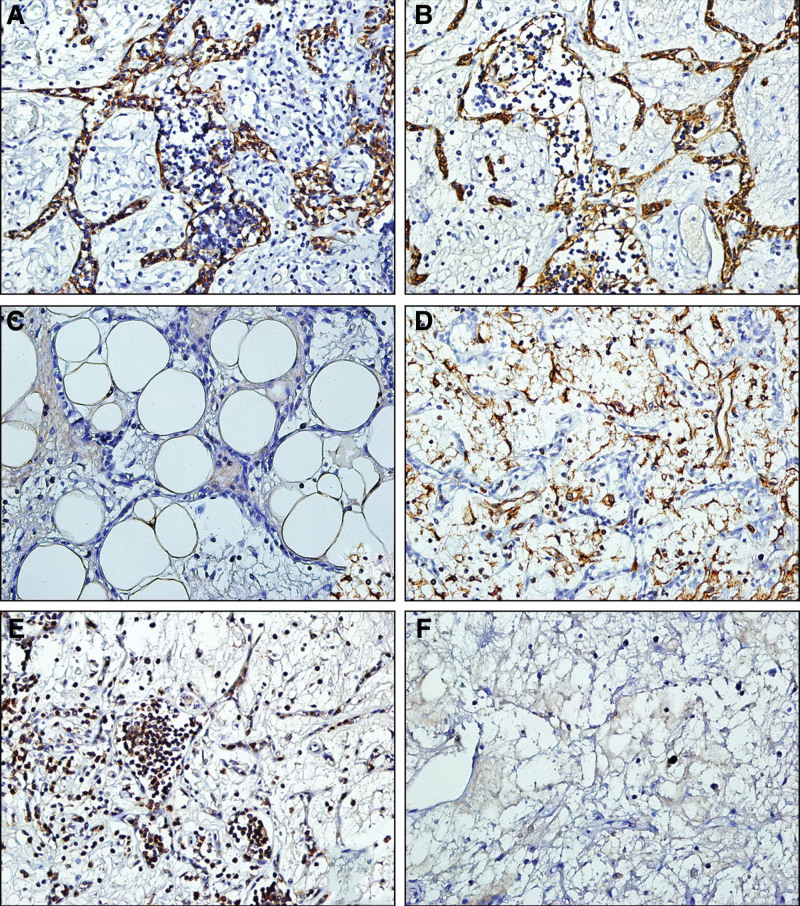
Immunohistochemical features of lipofibroadenoma. (Envision × 400) (A&B) The epithelial strands of the tumor are positive for CK19 and AE1/AE3. (C) the fat tissue is positive for S-100. (D) The fibrous tissue is positive for vimentin. (E) Lymphocytes are positive for CD3. (F) The Ki67 labeling index is calculated to be approximately less than 5%.

The EnVision 2-step method was used for immunohistochemical staining. All primary antibodies were provided by Zhongshan Golden Bridge Company. The epithelial cells of the tumor were positive for AE1/AE3 and CK19, the lymphocytes were positive for CD3 and CD20, and the fat and fibrous tissue were positive for S-100 and vimentin, respectively. The Ki67 labeling index was approximately less than 5% (Fig. 3). Based on histological features and immunophenotype, thymic lipofibroadenoma was diagnosed.

### 2.3. Follow-up

The patient was followed up 1 year after the operation, and no recurrence or residual lesions were found on the X-ray reexamination.

## 3. Discussion

Lipofibroadenoma is an unusual thymic tumor, and the clinical and pathological features remain unclear. Lipofibroadenoma is described as a “benign tumor” in the WHO classification of lung and thymus tumors (4th edition).^[5,6]^ To date, few cases have been reported. The first case was reported by Moran in 1994,^[7]^ and then, a few cases were reported in Britain, Japan, India, Turkey and China in succession (Table 1). All tumors were located in the anterior mediastinum with clear solid and fatty masses. All patients received thymectomy, and all were alive without evidence of disease after surgery.

**Table 1:** Summary of case reports of lipofibroadenoma.

Case	Age/Sex	Tumor size	Symptoms	Associated symptoms	Treatment	Follow-up results	Reference
1	62M	Not given	Dyspnea dizziness	Simple red blood cell regeneration disorder	Thymectomy	No recurrence	Kuo, T^[1]^
2	23F	21 × 7 × 5 cm	Chest pain dyspnea	Type B1 thymoma	Thymectomy	No recurrence	Aydin, Y^[2]^
3	20M	23 × 14 × 5 cm	Cough fever Night sweats	no	Thymectomy	No recurrence	Makdisi, G^[3]^
4	32M	Anterior mediastinum	No tumor-related symptoms	no	Thymectomy	No recurrence	Moran, C. A.^[7]^
5	28M	4.3 × 4.0 × 0.9 cm	No tumor-related symptoms	teratoma-like calcification	Thymectomy	No recurrence	Hakiri, S.^[8]^
6	29M	5.4 × 6.5 × 2.4 cm	Cough expectoration	Type B1 thymoma	Thymectomy	No recurrence	Hui, M.^[9]^
7	61M	8 × 7 × 3 cm	Myasthenia gravis	NO	Thymectomy	No recurrence	Anbardar, M. H.^[10]^
8	21M	8 × 6 × 2 cm	Fever	NO	Thymectomy	No recurrence	Current case

The primary site of lipofibroadenoma was located in the anterior mediastinum; however, it has also been described in the neck, pleura, or lung during radiological examination. The tumor usually appears radiographically as a smooth-edged lesion. As in the current study, patients usually experience localized symptoms such as cough, dyspnea, and pain. Lipofibroadenoma is often associated with myasthenia gravis (MG) and is rarely associated with hypogammaglobulinemia or pure red cell aplasia.^[1,11]^ In our case, lipofibroadenoma was diagnosed by chance, and the clinical symptoms were atypical or absent. Lipofibroadenoma rarely invades the pleura, pericardium, great vessels, or adjacent organs, and other distant metastases are extremely rare.^[12,13]^ In the present case, there was no invasion of mediastinal fat tissue. For lipofibroadenoma, complete surgical resection is possible in nearly all cases with less than 10% recurrence. The 10-year survival rate is higher than 90% for stage 1 or 2 disease. Staging is the most important prognostic factor. Age, sex, and other immunological diseases are not significant prognostic factors.^[4]^

This case was a young male patient with atypical clinical or absent symptoms. CT images showed space-occupying lesions on the left side of the anterior mediastinum, without paraneoplastic syndromes such as MG and simple red cell aplastic anemia. Pathological examination showed typical histological features of thymic lipofibroadenoma. In particular, the histological characteristics and immunophenotype of the tumor play an important role in the diagnosis and differential diagnosis.

The differential diagnosis of lipofibroadenoma by histology mainly included thymolipoma and sclerosing thymoma. Thymolipoma is an unusual thymoma that can lead to MG and autoimmune dysfunction. A recent report suggested that thymolipoma originates from true thymic hyperplasia. The report suggested that thymolipoma may be related to thymoma.^[14,15]^ Microscopically, epithelial and fibrous components cannot be observed in thymolipoma, which is an important point distinguishing it from lipofibroadenoma. In addition, biomarkers, including CD57, c-Jun, p73, Casp9, and N-ras, are also useful in the differential diagnosis.^[16,17]^

Sclerosing thymoma is a rare tumor that has the characteristics of traditional thymomas but with an abundant collagenous sclerosing matrix. The neoplasms are composed of lymphocytes mixed with scattered epithelial cells, and thymosomal structures can be seen. The tumor cells are nested and stringed into the transparent sclerosing stroma, which is the dominant component of the tumor. The main distinguishing point between sclerosing thymoma and lipofibroadenoma is the lack of mixed adipose tissue and fibrous tissue in the former.^[5]^

In summary, lipofibroadenoma is a rare and benign thymic tumor, and thymectomy is regarded as the best treatment. Currently, the understanding of this tumor remains at the histopathological level, and further research is needed to determine whether there are molecular genetic changes.

## Author contributions

FJ and CXW contributed equally to this work; FJ and CXW collected the clinicopathologic data and wrote the manuscript; HSY and LT collected and analyzed the radiological data; LXL was responsible for literature search and proofreading. All authors have read and approve the final manuscript.

**Conceptualization:** Xing-Lan Li.

**Data curation:** Shuang-Ye Hu, Tao Lu.

**Funding acquisition:** Xing-Lan Li.

**Project administration:** Xing-Lan Li.

**Writing – original draft:** Jing Fu, Xing-Wei Cai.

**Writing – review & editing:** Xing-Lan Li.
